# Unusual case of carcinoma cuniculatum affecting the oral cavity of an 11-year-old boy^⋆^^⋆⋆^

**DOI:** 10.1016/j.abd.2020.08.029

**Published:** 2021-09-25

**Authors:** Sven Niklander, Luz María Sernuda, René Martinez

**Affiliations:** a Department of Surgery and Oral Pathology, Faculty of Dentistry, Universidad Andres Bello, Santiago, Chile; b Maxillofacial Surgery, Quilpué Hospital, Viña del Mar-Quillota Health Service, Quilpué, Chile

Dear Editor,

Oral Carcinoma Cuniculatum (OCC) corresponds to a rare, well-differentiated variant of Oral Squamous Cell Carcinoma (OSCC) first included in the World Health Organization (WHO) Classification of Head and Neck tumors in 2005. According to the WHO, OCC is defined as a malignant neoplastic proliferation of stratified squamous epithelium in broad processes with keratin cores and keratin-filled crypts which seem to burrow into the bone, without obvious cytological features of malignancy.^1^ It is estimated that OCC accounts for up to 2.7% of all OSCC.^2^ Up to date, less than 60 cases have been reported. Here we report an unsual case of an oral carcinoma cuniculatum affecting the gingiva of a child.

An 11-year-old boy was consulted because of local pain and paresthesia of the left lower lip during an unknown amount of time. He had a history of grade II ependymoma, which was appropriately treated with surgery and radiotherapy and controlled routinely. He had no systemic symptoms, known allergies or medical history of any other illness. Extra-oral examination revealed no anomalies. Intraorally, a mass involving both the buccal and lingual gingiva between the lower left second molar and lower left first premolar was observed. An extensive verrucous white patch was covering the buccal side of the mass, while cystic-like white nodules with superficial blood vessels were also present on both the lingual and buccal sides of the tumor (Fig. 1). The teeth involved in the mass were mobile. Cone-beam computed tomography revealed a well-defined large radiolucency involving the whole thickness of the posterior left mandible perforating the buccal bone plate (Fig. 2). An incisional biopsy was taken which showed a tumoral mass composed of multiple branching keratin-filled crypts sheets lined by epidermoid epithelial cells. The endophytic epithelial growth pattern burrowed deep inside into the alveolar bone, forming a complex network of connected canaliculi, resembling rabbit burrows. The epithelial cells were well differentiated, and no atypia, mitosis, perineural nor lymphovascular invasion was observed. A chronic inflammatory infiltrates consisting mainly of lymphocytes with local areas of neutrophil accumulation was observed in some areas of the stroma (Fig. 3). The lesion was diagnosed as a carcinoma cuniculatum of the oral cavity. Differential diagnosis with a solid variant of an odontogenic keratocyst was done due to its histopathological similarity. The CT scan examination revealed no lymph node involvement and no metastasis, and the patient was referred for surgical treatment. The patient was controlled with regularity and no recurrences have been observed after a 5-years-follow up period.

**Figure 1:**
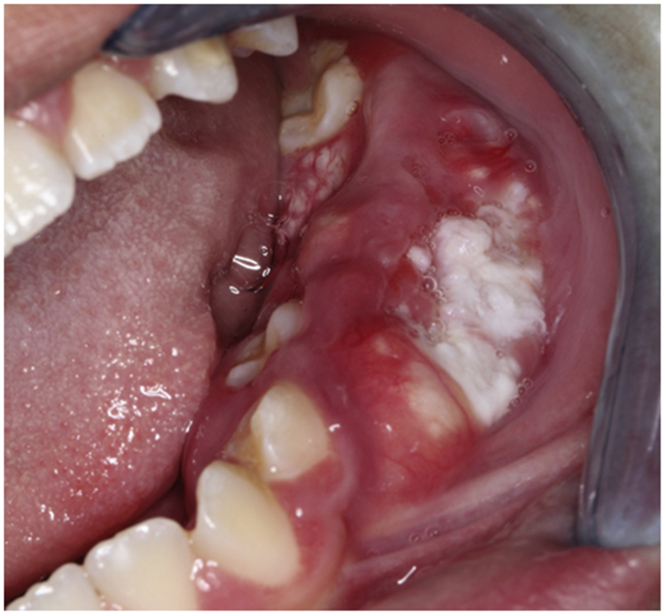
Clinical presentation: mass involving both the buccal and lingual gingiva between the lower left second molar and lower left first premolar.

**Figure 2:**
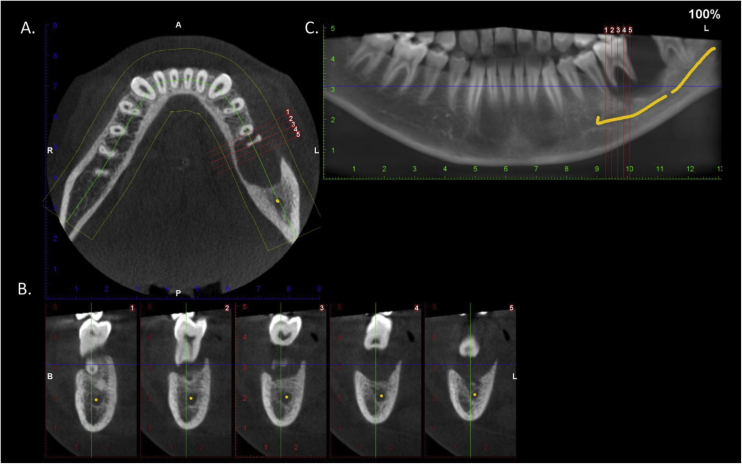
Cone-beam computed tomography. Axial (A) and para-axial (B) sections and panoramic reconstruction (C). There is a well-defined large radiolucency involving the whole thickness of the posterior left mandible perforating the buccal bone plate.

**Figure 3:**
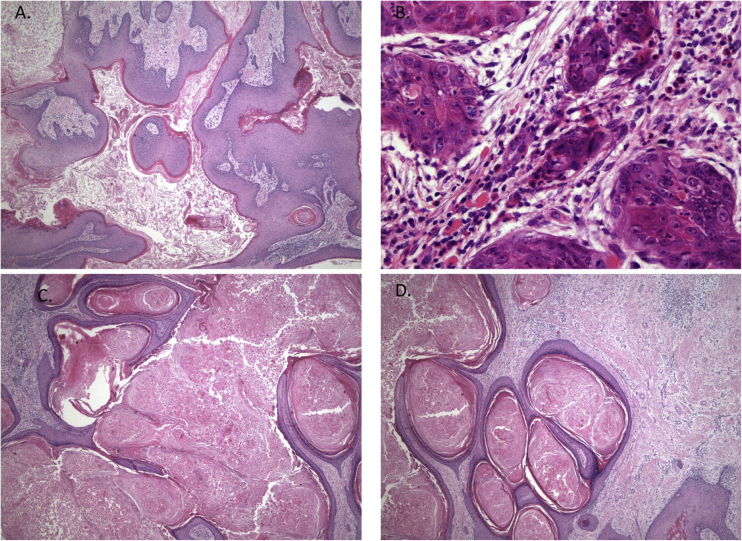
Histopathological features showing neoplastic tissue with an endophytic pattern of growth with abundant keratinization (A). A mixed inflammatory infiltrate composed primarily of lymphocytes and some neutrophils were seen in some areas (B). The endophytic epithelial growth was forming a complex network of connected canaliculi resembling rabbit burrows (C and D). Original magnification was ×10 (A, C and D) and ×40 (B).

Carcinoma Cuniculatum (CC) is a rare, low-grade variant of Squamous Cell Carcinoma (SCC) most commonly located on the plant of the foot, but any anatomic site can be affected, including the oral cavity.^2^ According to a recent systemic review that analyzed 43 cases of OCC, this tumor has a slight predilection for females, being more frequent between the sixth and seventh decade of life.^3^ Our case is the second report of an OCC in children. The first case was reported by Hutton el al. in 2010, which corresponded to an OCC affecting the anterior maxillary gingiva of a 7-year-old girl.^4^ When affecting the oral cavity, the most common location is the mandibular gingiva followed by the maxillary gingiva.^3^ Cases affecting the alveolar ridge, tongue and palate have also been reported.^5^ When affecting the gingiva, bone involvement is common. Imaging usually shows a radiolucency with ill-defined margins reabsorbing the adjacent cortical bone.^6^

The most common clinical symptom is pain, followed by ulceration, swelling, and induration.^3^, ^5^, ^6^ Our patient apart from the aforementioned symptoms also reported paresthesia, which to our knowledge, has not been reported before. The main differential diagnosis of CC is Verrucous Carcinoma (VC), another well-differentiated uncommon variant of SCC. But other keratinizing lesions, such as keratinizing odontogenic cysts have to be ruled out, especially with dealing with small incisional biopsies. In the early days, CC was thought to be a VC, but is now considered a distinct entity of SCC.^2^ Clinically, both CC and VC can have a verrucous appearance, although CC might exhibit a sessile pink-reddish papillary surface, which is not observed in VC. Histologically, CC shows a tortuous invasive (endophytic) component with keratin plugging, which contrasts with the more exophytic verrucous growth and “pushing borders” observed in VC.^5^ Microabscesses filled with neutrophils are also commonly reported in CC.^2^

The main treatment of choice is surgical excision. Although OCC can be locally aggressive, lymph node and distant metastases are uncommon,^2^, ^6^ and the 5-year survival rate has been reported above 90%. ^2^ Local recurrences have been reported, but are not very common. In respect to our case, the patient remained disease-free after a follow-up period of 5 years.

## Financial support

None declared.

## Authors' contributions

Sven Niklander: Preparation and writing of the manuscript; approval of the final version of the manuscript.

Luz María Sernuda: Approval of the final version of the manuscript.

René Martinez: Preparation and writing of the manuscript; approval of the final version of the manuscript.

## Conflicts of interest

None declared.

This research did not receive any specific grant from funding agencies in the public, commercial, or not-for-profit sectors. The study was done in Viña del Mar, Chile.
